# Supplementary material for: The Triad Na+ Activated Na+ Channel (Nax)—Salt Inducible KINASE (SIK) and (Na+ + K+)-ATPase: Targeting the Villains to Treat Salt Resistant and Sensitive Hypertension
Source: Int J Mol Sci. 2023 Apr 26;24(9):7887. doi: 10.3390/ijms24097887 (PMC10178781; doi:10.3390/ijms24097887)
Supplement: Supplementary file 1 [file ijms-24-07887-s001.zip › ijms-2230446-supplementary.pdf]

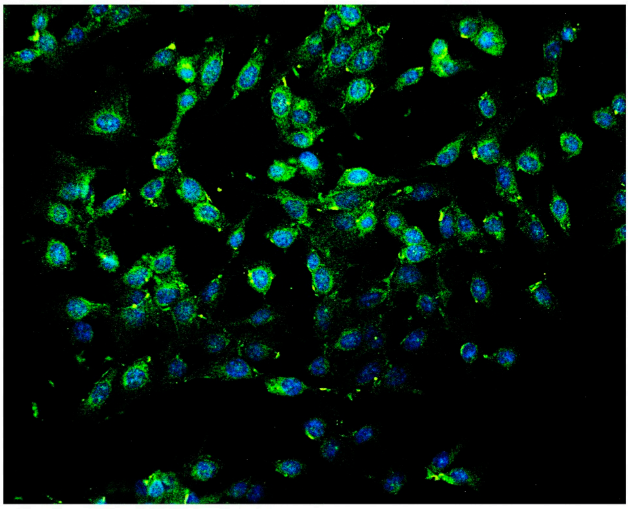

Supplementary Figure S1. Salt inducible kinase 1 (SIK) detection in rat proximal tubule cells (RPT cells) by immunofluorescence. Immortalized rat proximal tubule cells (RPT) were kindly provided by Dr. Ingelfinger (Harvard Medical School) and used in this study [1]. RPT cells were cultured in four-well chambers (Lab-Tek). Immunofluorescence was performed according to [2]. The cell culture media was removed, then rinsed with PBS and fixed for 20 min by 4% paraformaldehyde. After 4 minutes of incubation with 0.2% Triton X-100, the blocking agent Image-iT FX signal enhancer (Invitrogen, CA, USA) was added to the chambers. The fixed cells were incubated with primary rabbit SIK1 polyclonal antibody (1:500; sc-83754; Santa Cruz Biotechnology) for 2 h. After washing with PBS, ProLong Gold antifade reagent with DAPI (Invitrogen) was used as a nuclear stain and a mount reagent. RPT cells were observed and photographed under a fluorescence microscope (Olympus BX51, Olympus Optical Co. Ltd.).

References

1. Ingelfinger, J.R.; Jung, F.; Diamant, D.; Haveran, L.; Lee, E.; Brem, A.; Tang, S.S. Rat proximal tubule cell line transformed with origin-defective SV40 DNA: autocrine ANG II feedback. *Am. J. Physiol.* **1999**, *276*, F218–F227.
2. Garagliano, J.M.; Katsurada, A.; Miyata, K.; Derbenev, A.V.; Zsombok, A.; Navar, L.G.; Satou, R. Advanced Glycation End Products Stimulate Angiotensinogen Production in Renal Proximal Tubular Cells. *Am. J. Med. Sci.* **2019**, *357*, 57–66. https://doi.org/10.1016/j.amjms.2018.10.008.
